# Fluid Biomarkers of Cognitive Impairments Following Traumatic Brain Injury: A Systematic Review and Meta Analysis

**DOI:** 10.3390/ijms27104274

**Published:** 2026-05-11

**Authors:** Yingdi Liao, Lianna Zhao, Youyang Zhu, Sirong Ye, Jinqing Huang, Zhichao Niu, Luoqing Zhang, Na Lei, Peixin Guo, Yuhuan Xie

**Affiliations:** 1 First Clinical Medical College, Yunnan University of Chinese Medicine, Kunming 650500, China; ying19961215@126.com (Y.L.); zyy980213@163.com (Y.Z.); 2 School of Chinese Materia Medica, Yunnan University of Chinese Medicine, Kunming 650500, China; cjs48ws@163.com (L.Z.); 15187471928@163.com (S.Y.); 15677588716@163.com (J.H.); 15126599975@163.com (L.Z.); 3 School of Basic Medical Sciences, Yunnan University of Chinese Medicine, Kunming 650500, China; 15650079634@163.com (Z.N.); kmleina3328@126.com (N.L.); 4 The College Based Key Laboratory of Yunnan in Aromatic Chinese Herbal Research, Kunming 650500, China; 5 College of Ethnic Medicine, Yunnan University of Chinese Medicine, Kunming 650500, China; 6 Science and Technology Department, Yunnan University of Chinese Medicine, Kunming 650500, China

**Keywords:** traumatic brain injury, fluid biomarkers, cognitive impairment, Alzheimer’s disease, systematic review and meta-analysis

## Abstract

Traumatic brain injury (TBI), a major cause of persistent cognitive impairment (CI), increases the long-term risk of developing dementia, including Alzheimer’s disease (AD). To elucidate this association, we systematically reviewed fluid biomarkers linked to post-TBI cognitive outcomes. A comprehensive search of the PubMed, Embase, and Cochrane Library databases was performed. A total of 29 clinical studies were included, reporting on several biomarkers related to neural injury and repair, AD-like pathology, and inflammation. Among these, neurofilament light chain (NfL), ubiquitin C-terminal hydrolase L1, total tau, and glial fibrillary acidic protein (GFAP) were consistently associated with CI and brain atrophy across various TBI severities and stages. Notably, certain biomarkers assessed during the acute phase (within 7 days post-injury), such as brain-derived neurotrophic factor, neuron-specific enolase, and interleukin-1β, showed significant correlations with CI. In contrast, elevated levels of GFAP and NfL measured during the recovery phase (6 months to 8 years post-injury) were significantly associated with TBI-related CI (TBI-CI). The findings also highlighted that axonal injury, glial activation, neuroinflammation, neuronal damage, and degeneration drive TBI-CI, with tau pathology and synaptic dysfunction emerging as potential bridges from TBI to AD. This review underscores the critical temporal dynamics of fluid biomarkers in TBI-CI, revealing that stage-specific biomarker profiles mirror distinct underlying pathophysiological processes. Future longitudinal studies should focus on well-characterized patient subgroups, adopt standardized diagnostic criteria, and integrate fluid biomarkers with neuroimaging and genetic data.

## 1. Introduction

Traumatic brain injury (TBI), defined as structural damage or functional impairment of the brain resulting from external mechanical forces to the head, is a leading global cause of mortality and disability, posing a significant health burden [1,2]. With an estimated annual incidence exceeding 50 million cases worldwide [3], TBI represents an escalating public health concern [4]. It can affect individuals across all age groups and is commonly caused by traffic accidents, falls, contact sports, occupational injuries, or violent incidents [5]. TBI can lead to various neurological and cognitive consequences, including motor deficits, cognitive impairment (CI), traumatic epilepsy, and personality changes [2]. Among these, CI is one of the most persistent and common symptoms, with cohort studies reporting its occurrence in 20.2–72.5% of TBI survivors long-term [6,7].

Extensive studies have documented the spectrum of CI following TBI, spanning from the acute to the chronic stages [8,9]. These impairments encompass deficits in executive function, episodic memory, attention, working memory, decision-making, and reaction time [8]. Although some patients experience cognitive improvement over time, individuals with moderate or severe TBI (sTBI) frequently suffer from persistent cognitive decline, which may gradually progress into various forms of dementia over the years [10]. Supporting this finding, longitudinal studies have revealed that brain atrophy frequently develops in patients with TBI months to years following injury, and such structural degeneration may further exacerbate cognitive decline [11]. Epidemiological evidence further indicates a long-term association between TBI and dementia. Patients with a history of TBI are at a high risk of developing dementia later in life [relative risk (*RR*) = 1.63, 95% confidence interval (*CI*): 1.34–1.99] [12,13], with a particularly strong association for developing Alzheimer’s disease (AD) (*RR* = 1.51, 95% *CI*: 1.26–1.80) [12]. This specificity may be attributed to the AD-like pathological changes triggered by TBI. Postmortem pathological studies have revealed that approximately 30% of patients who die acutely post-TBI exhibit amyloid-beta (Aβ) plaques [14] and neurofibrillary tangles composed of phosphorylated tau (P-tau) [15], both hallmark features of early-stage AD.

Current evidence suggests that the mechanisms underlying TBI-related CI (TBI-CI) are highly complex, involving acute axonal injury [16], neuronal damage [17], endothelial cell and blood–brain barrier damage [18], and persistent inflammation [19]. These processes may synergistically accelerate complex pathological deposition, leading to the occurrence of dementia. As CI is a common and core symptom of AD and other forms of dementia [20,21], increasing attention has been directed toward uncovering the neuropathological mechanisms of TBI-CI. The early identification and continuous monitoring of high-risk patients with TBI are crucial for informing clinical decision-making and improving prognoses.

Consequently, fluid biomarkers provide a critical window into the early detection and longitudinal progression of TBI-CI, offering valuable insights into underlying pathophysiological mechanisms and enabling the development of predictive models over time. A review published in 2014 identified several biomarkers associated with TBI-CI and highlighted the significance of neurotransmitter dysfunction in cognitive deficits. However, its findings were primarily derived from animal studies, with limited clinical evidence available at the time [22]. In recent years, a growing number of clinical studies have investigated novel biomarkers linked to TBI-CI [23,24,25], such as tau proteins and neurofilament light chain (NfL). However, existing evidence remains fragmented, and a systematic integration of cerebrospinal fluid (CSF) and blood-based biomarker data is lacking, particularly regarding the assessment of their clinical validity in predicting the trajectory of TBI-CI. Previous reviews have summarized biomarker–cognition associations [22] but share several important limitations: (1) they predominantly focus on single or cross-sectional time points, with limited consideration of temporal dynamics from the acute to chronic phases; (2) they lack systematic stratification by injury severity.

In response to these gaps, this study provides a systematic synthesis integrating CSF and peripheral blood biomarker evidence across three key dimensions: temporal trajectory, disease stage, and injury severity. We examine the associations and potential prognostic relevance of these fluid biomarkers in relation to TBI-CI, with particular emphasis on markers showing relatively consistent longitudinal associations, providing novel insights into underlying mechanisms and supporting early detection and precision medicine strategies.

## 2. Methods

This systematic review was performed according to the Preferred Reporting Items for Systematic Reviews and Meta-Analyses (PRISMA) guidelines [26] and the protocol is registered in PROSPERO (CRD42024603840).

### 2.1. Search Strategy and Inclusion Criteria

Three English databases (PubMed, Embase, Cochrane Library) were comprehensively searched from their inception until August 2024. The search was restricted to studies published in English. The search terms included “traumatic brain injury”, “head injury”, “cognitive impairment”, “Alzheimer’s disease”, and “biomarkers”. The detailed search strategies for each database are provided in the Supplementary Materials. Observational clinical trials were included, encompassing cross-sectional, cohort and case–control studies.

The included studies involved adult patients with TBI diagnosed according to established criteria [27]. Cognitive function was assessed or CI was diagnosed during follow-up using standardized methods, including neuroimaging and validated cognitive scales and various neuropsychological tests. Control groups comprised patients without TBI-CI or healthy individuals. Exclusion criteria included case reports, reviews, meta-analyses, animal studies, studies involving other neurological or psychiatric disorders, and studies that did not assess post-TBI fluid biomarkers or cognitive outcomes. Studies were excluded if they were non-adult research (participants < 18 years old). Studies focusing on hormonal or endocrine biomarkers were also excluded.

### 2.2. Study Selection and Data Collection

Two reviewers (YL and YZ) independently screened titles and abstracts after duplicate removal, followed by full-text eligibility assessment. Discrepancies were resolved through discussion. Data extraction was independently performed by two reviewers (YL and LZ) using pre-designed forms, with cross-checking for accuracy. Extracted data included study characteristics, participant characteristics, and outcomes. Numerical data presented graphically were extracted using the GetData software (GetData Graph Digitizer version 2.25.0.32). Any disagreements were resolved by consultation with a third reviewer (YX).

### 2.3. Risk of Bias Assessment

Two authors (YL and SY) independently evaluated the risk of bias in the included studies using the Newcastle–Ottawa Scale (NOS) [28]. The assessment covered selection, comparability, exposure ascertainment, and outcome assessment, with total scores ranging from 0 to 9. Disagreements were resolved by discussion or adjudication by a third reviewer (YX).

### 2.4. Data Synthesis

All statistical analyses were performed using Review Manager software (RevMan, Version 5.3). For continuous outcomes, results were expressed as mean differences (*MD*) with 95% *CI*, whereas dichotomous outcomes were presented as RR with 95% *CI*. When studies reported data as medians with interquartile ranges (IQRs) or ranges, these values were converted to means and standard deviations using the method described by McGrath et al. [29]. In cases where biomarker detection methods or sources varied substantially across studies, standardized mean differences (*SMD*) were calculated to enable data pooling. Heterogeneity was assessed using *I*^2^, guiding the choice between fixed- and random-effects models. Subgroup and descriptive analyses were conducted to explore heterogeneity. Publication bias for the biomarkers was assessed only when at least 10 studies were included, as the statistical power of these methods is limited with fewer studies. In such cases, funnel plots were used for qualitative assessment of symmetry, followed by Egger’s test for a quantitative evaluation.

## 3. Results

### 3.1. Literature Search Findings

**Figure 1:**
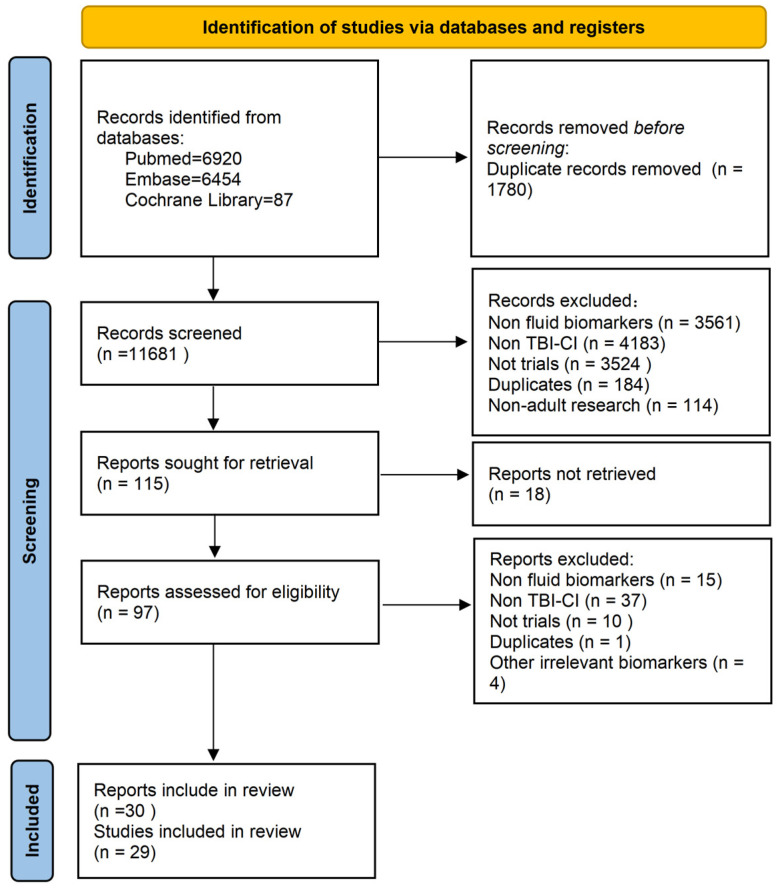
PRISMA flow diagram.

In this systematic review and meta-analysis, 29 studies [23,24,25,30,31,32,33,34,35,36,37,38,39,40,41,42,43,44,45,46,47,48,49,50,51,52,53,54,55,56], published in 30 articles, were included after screening titles, abstracts, and full texts from the three databases. Among these, the studies by Neil Graham in 2021 [30] and 2023 [31] were identified as the same study reported in different journals. The process of study selection and screening is illustrated in Figure 1.

### 3.2. Characteristics of the Included Studies and Risk of Bias Assessment

The included studies were published between 2005 and 2024, with 22 (75.8%) being published after 2020. Study designs comprised 19 cohort studies, 6 case–control studies, and 4 cross-sectional studies, with sample sizes ranging from 30 to 472 participants. Only two specifically reported AD following TBI [32,33], while the remainder focused on TBI-CI. Regarding disease phase, 10 studies assessed the acute phase (≤7 days post-injury) [37,38,47,50,51,52,53,54,55,56], 3 evaluated the subacute phase (7 days to 1 month post-injury) [30,31,34], and 13 reported the chronic phase (typically more than 1 month post-injury) [23,24,25,32,33,35,39,42,43,44,45,46,48]. The included studies represented a range of TBI severities and populations, with 9 studies specifically investigating mild TBI (mTBI) [34,41,47,50,52,53,54,55,56], and 5 focusing on moderate to sTBI [30,35,36,37,38]. In addition, 2 studies focused on repetitive head injury [23,39], and 6 studies were conducted in veteran populations [24,40,41,42,43,44].

Identified biomarkers were categorized as neural injury or repair markers, AD-related pathological markers, and inflammatory or cytokine markers. The most frequently reported biomarkers were NfL, total tau (T-tau), glial fibrillary acidic protein (GFAP), ubiquitin C-terminal hydrolase-L1 (UCH-L1), P-tau 181, Aβ42, interleukin-1*β* (IL-1*β*), and S100 calcium-binding protein B (S100B), measured mainly in serum, plasma, or CSF using enzyme-linked immunosorbent assay (ELISA) or single-molecule array (Simoa). Cognitive outcomes were evaluated using a comprehensive range of neuropsychological tests and imaging modalities, with four studies additionally incorporating volumetric analyses based on structural magnetic resonance imaging (MRI) [30,31,45,46]. Methodological quality was evaluated using the Newcastle–Ottawa Scale (NOS). Scores ranged from 4 to 8, with two studies scoring 8, eleven scoring 7, and approximately 53.3% of studies were rated as moderate-to-low quality (≤6). Detailed study characteristics and quality assessments are summarized in Table 1.

### 3.3. Associations Between Fluids Biomarkers and Cognition Following TBI

#### 3.3.1. Neuro-Injury Markers

Multiple studies identified neural injury-related biomarkers associated with cognitive function in patients with TBI, including NfL, UCH-L1, neuron-specific enolase (NSE), spectrin N-terminal fragment (SNTF), and neuronal pentraxin 2 (NPTX2). These neuronal biomarkers are primarily derived from axons and neuronal cell bodies, and are released in response to structural damage and cytoskeletal disruption.

##### Axonal Damage Markers: NfL and SNTF

NfL, a key structural component of the axonal cytoskeleton, is primarily expressed in large myelinated axons but is also detectable in somatic cells and dendrites, reflecting its broader involvement in maintaining neuronal integrity [57]. The structural breakdown of axons leads to the release of NfL, which is detectable in both CSF and blood, though the former typically contains ten times the concentration of the latter [58].

Eleven studies explored the relationship between fluid NfL levels and CI or brain atrophy after TBI [23,24,25,30,32,39,40,45,46,48,56]. Meta-analysis demonstrated significantly elevated serum or plasma exosomal NfL levels in chronic-phase TBI-CI compared with TBI-no-CI patients [24,48] (*SMD* = 0.89, 95% *CI*: 0.55–1.22, *n* = 166, *I*^2^ = 23%, *p* < 0.001, Figure S1), with moderate discriminative ability for distinguishing TBI-CI from TBI-no-CI [area under the curve (AUC) = 0.76, 95% *CI*: 0.64–0.89, *p* = 0.01 [24]. Several studies also reported that plasma and serum NfL levels were associated with cognitive function and brain structural changes [25,30,39,40], as well as a predictive value for long-term white matter, gray matter, hippocampal, and thalamic atrophy (Table S1) [23,25,30,45,46,56]. However, two studies reported null associations [32,48], likely reflecting heterogeneity in cognitive assessment tools and sampling time points. One study showed that a slower rate of decline in serum NfL levels from 30 days to 5 years post-TBI was associated with a faster rate of brain volume loss [46].

The αII-spectrin N-terminal fragment (SNTF, αII-spectrin breakdown products) is a stable breakdown product derived from the αII-spectrin that is abundant in axons and presynaptic terminals [57]. SNTF serves as a marker of calpain-mediated axonal degeneration, particularly following acute neurological insults such as TBI or cerebral ischemia [57]. A recent cohort study demonstrated a significant association between plasma SNTF levels and cognitive performance and recovery in patients with mTBI (*p* < 0.025) [50].

##### Neuron Cell Body Damage: NSE, UCH-L1

NSE, an enzyme uniquely expressed in neuronal cell bodies, is a clinically validated prognostic marker for cerebral hypoxia [59]. NSE demonstrated prognostic value in a longitudinal study, with early serum levels predicting short- and medium-term CI [37] (Table S1).

As a neuron-specific deubiquitinase enriched in the neuronal cytoplasm, UCH-L1 is significantly elevated in blood and CSF during the acute phase of TBI [60] and has emerged as a potential biomarker for neuronal injury [61]. One study demonstrated that serum UCH-L1 levels were significantly higher in the TBI-CI group than in the TBI-no-CI group (*MD* = 0.12, 95% *CI*: 0.06–0.18, *n* = 101, *I*^2^ = 0%, *p* < 0.001) [48]. Several studies have identified correlations between serum UCH-L1 levels and post-TBI cognitive outcomes as well as brain volume [25,30,40,46,53] (Table S1). Baseline serum UCH-L1 levels were associated with information processing speed and working memory, and increased UCH-L1 levels within 1 year post-TBI were associated with declines in memory indices (*p* < 0.05, Table S1) [40]. Early post-injury UCH-L1 levels in serum also predicted CI at 6 months (*β* = −0.597, *p* < 0.001) [56], highlighting its potential as both a diagnostic and prognostic biomarker of TBI-CI.

##### Synaptic Damage Markers

Limited evidence suggests that synapse-derived biomarkers play a role in TBI-CI. NPTX2, a protein essential for excitatory postsynaptic homeostasis, showed reduced CSF levels associated with longitudinal attention declines in patients with AD and a history of TBI (*β* = −0.116, *p* = 0.045, Table S2) [32]. These findings may indirectly support a role of synaptic dysfunction in TBI-CI. Variations in synaptic marker levels may reflect dynamic processes of synaptic remodeling as well as the chronicity of TBI-related pathology. Additionally, one study reported elevated plasma exosomal levels of cellular prion protein (enriched in synaptic membranes) and synaptogyrin-3 (a vesicular membrane protein) in patients with TBI-CI (*p* < 0.01) [43], while plasma exosomal *α*-synuclein (enriched in presynaptic terminals) levels showed no significant difference between the TBI-CI and TBI-no-CI groups [24].

#### 3.3.2. Glial Cell Damage Markers: GFAP and S100 Proteins

GFAP, a class III intermediate filament protein and major structural component of astrocytes, is predominantly expressed in reactive astrocytes and serves as a specific biomarker for astrogliosis, which often surrounds Aβ plaques in AD [62]. Meta-analysis revealed significantly higher serum or plasma exosomal GFAP levels in TBI-CI patients (*SMD* = 0.88, 95% *CI*: 0.54–1.21, *n* = 166, *I*^2^ = 23%, *p* < 0.001, Figure S1) [24,36,48], with moderate discriminative ability between the TBI-CI and TBI-no-CI groups (AUC = 0.71, *p* = 0.04) [24]. Elevated GFAP levels following TBI were significantly associated with impairments in executive function and processing speed, as well as reduced gray and white matter volumes [23,25,30,39,40,46] (Table S3).

As the key astroglial injury biomarkers in TBI management [47,63], S100B and S100A1 are associated with cognitive outcomes, yet the vast majority of this evidence is derived from serum samples rather than CSF. Two studies reported that elevated serum S100B levels measured during the acute- and subacute-phase post-TBI were significantly associated with poorer performance in working memory, verbal learning, and verbal fluency at 3-month follow-up [53], and prospectively predicted gray matter atrophy at 6 months [30] (Table S3). The ROC curve analysis indicated its predictive utility for cognitive outcomes, with an AUC of 0.704 (*p* < 0.05) [49]. However, several studies reported null associations, suggesting that the predictive value of S100B may be time-dependent and context-specific [37,47].

#### 3.3.3. Markers Related to Neural Plasticity and Repair

Two studies identified neural plasticity and repair biomarkers associated with cognitive function in patients with TBI, including neural cell adhesion molecule (NCAM) and brain-derived neurotrophic factor (BDNF). As a key neurotrophin involved in neural regeneration [64], BDNF, which is highly expressed in neurons, has been implicated in memory and learning, as well as TBI pathophysiology [65]. Notably, acute-phase BDNF levels were positively associated with long-term cognitive outcomes, particularly memory, whereas chronic-phase serum or CSF BDNF showed no such association [38] (Table S2), suggesting a time-dependent relationship. In parallel, serum NCAM, which mediates neuron-neuron interaction and regulates synaptic plasticity [66], was significantly elevated in TBI patients with CI compared to those without [36].

#### 3.3.4. Pathological Biomarkers Related to AD: Tau Proteins and Aβ Peptides

Tau is a versatile microtubule-associated protein whose biofluid concentrations are established indicators of disease severity in neurodegeneration [67,68]. In neurodegenerative conditions such as AD, the phosphorylation of Tau leads to the formation of toxic aggregates and a concomitant loss of its physiological function [67,69].

However, existing studies have not provided consistent evidence for its value as a biomarker for TBI-CI. Meta-analysis showed that CSF T-tau levels were marginally higher in patients with AD and a history of TBI than in those without TBI (*MD* = 81.2, 95% *CI*: 1.45–160.94, *n* = 243, *I*^2^ = 0%, *p* = 0.05, Figure S2) [32,33]. Following axonal damage, tau undergoes proteolytic cleavage by activated caspases and calpains, yielding peptide fragments that are detectable in peripheral blood. So several studies suggested the predictive value of blood T-tau for long-term outcomes, showing that elevated serum and plasma T-tau levels within 6 weeks post-injury predicted long-term loss in gray matter volume [30,46]; moreover, serum levels measured on days 1, 3, and 5 post-injury correlated with CI at 6 months [51], and baseline levels at 1 year post-injury were associated with greater declines in executive function over time [40] (Table S2). However, most studies reported no significant associations between fluid T-tau levels and post-TBI cognitive outcomes [25,32,41,44,48], and no consistent differences were observed between TBI-CI and TBI-no-CI groups [24,48].

Evidence for P-tau was similarly heterogeneous. In an observational study of veterans, plasma exosomal P-tau 181 and P-tau 396 were significantly elevated in individuals with CI compared to those without, persisting several years post-injury. Pearson correlation analysis revealed significant inverse relationships between exosomal P-tau 181 levels and cognitive performance scores (*p* < 0.05) [43]. Consistent with this, elevated CSF P-tau levels were linked to CI in selected chronic TBI cohorts [44] (Table S1). Conversely, pooled analyses showed no significant differences in CSF P-tau181 levels between AD patients with and without a history of TBI (*MD* = 7.29, 95% *CI*: −0.77–15.36, *n* = 243, *I*^2^ = 0%, *p* = 0.08, Figure S2) [32,33]. Several studies also reported no significant associations between P-tau levels, irrespective of the specific phosphorylation epitope assessed, and post-TBI cognition outcomes or brain volumes [23,31,41].

Aβ is a peptide derived from the proteolytic cleavage of amyloid precursor protein. While the dysregulation of Aβ is a key pathological feature of AD [67,70], its role in TBI is increasingly recognized. Specially, reduced CSF Aβ peptide levels following TBI have been proposed as surrogate markers for Aβ deposition in the brain [71]. However, the relationship between Aβ and TBI-CI remains controversial. The pooled analyses showed no significant differences in CSF Aβ42 levels between AD patients with and without a history of TBI (*MD* = 1.04, 95% *CI*: −38.65–40.73, *n* = 243, *I*^2^ = 0%, *p* = 0.96, Figure S2) [32,33], and no consistent correlations with cognitive outcomes were observed [32,42,44]. Although individual studies reported associations between reduced CSF Aβ42 [41] or Aβ40 levels [42] and domain-specific cognitive deficits (Table S1), results regarding plasma exosomal Aβ42 remain inconsistent. One study reported elevated plasma exosomal Aβ42 in chronic TBI-CI patients [43], whereas another found no significant difference between TBI-CI and TBI-no-CI groups [24]. This inconsistency may reflect methodological and clinical heterogeneity, including differences in exosome isolation techniques, patient populations, and assay platforms used for biomarker measurement.

#### 3.3.5. Systemic Inflammatory Markers and Cytokines

Following TBI, the innate immune cells within the central nervous system, particularly microglia and astrocytes, are rapidly activated and contribute to secondary injury through the release of pro-inflammatory mediators [72]. Inflammatory biomarkers showed phase-dependent associations with cognitive performance.

In the acute phase, elevated serum and plasma IL-1*β* were consistently associated with short- and long-term CI [52,54,55,56], whereas no association was observed during the chronic phase (Table S4) [35]. IL-6 demonstrated predictive value for CI (AUC = 0.78, *p* = 0.004) [24,43] and was associated with worse cognitive outcomes [56], particularly in patients with concurrent Aβ pathology [39]. Similarly, elevated serum or plasma exosomal tumor necrosis factor *α* (TNF-*α*) levels during chronic phase were associated with long-term CI following TBI (*p* = 0.05, Table S4) [35], and showed discriminative ability for distinguishing TBI-CI from TBI-no-CI cases (AUC = 0.8, *p* = 0.003) [24]. In contrast, findings for other inflammatory mediators have been heterogeneous. Elevated plasma C-reactive protein (CRP) levels were significantly associated with TBI-CI (*OR* = 1.687, 95% *CI*: 1.135–2.507, *p* = 0.010) [34]. Other serum cytokines yielded mixed or domain-specific effects, with some markers demonstrating positive [52] and others negative associations with specific cognitive domains [35]. The detailed results are summarized in Table S4.

#### 3.3.6. Subgroup Analysis and Publication Bias

To evaluate the predictive value, discriminative ability, association of biomarkers for TBI-CI, subgroup analyses were conducted by injury severity and disease phase (Table 2). In mTBI, acute-phase plasma NfL, UCH-L1, and S100B, as well as CRP levels during the subacute phase, have been associated with long-term cognitive outcomes. In the chronic phase, a reduced CSF Aβ42 or Aβ40 levels is linked to CI, particularly in individuals aged ≥45 years in some cohorts, whereas findings for tau-related biomarkers remain inconsistent. In sTBI, acute-phase NSE, S100B, and BDNF have been associated with memory outcomes and recovery, while plasma NfL, GFAP, UCH-L1, NCAM, T-tau, and inflammatory markers have been associated with structural brain changes and cognitive decline, although considerable heterogeneity across studies has been observed. Notably, NfL, GFAP, and UCH-L1 demonstrate relatively consistent associations across studies with cognitive outcomes in both mTBI and sTBI cohorts, suggesting their potential utility as prognostic and predictive biomarkers for TBI-CI across different injury severities. In contrast, exosomal P-tau181, Aβ42, and several other biomarkers show promising but still preliminary evidence.

In addition, biomarkers from different biological sources were also summarized in Table 3. T-tau and P-tau have been reported to be associated with TBI-CI across multiple biological matrices, including blood, exosomes, and CSF, supporting the involvement of tau pathology in post-TBI cognitive decline. However, the associations of Aβ42 and BDNF with TBI-CI appear to be highly dependent on the type of biological sample analyzed, which may reflect differences in amyloid metabolism and neurotrophic signaling across compartments. Most biomarkers demonstrate statistical associations with cognitive outcomes rather than established predictive performance, and their clinical applicability should therefore be interpreted with caution.

As most biomarkers included in the quantitative analysis were based on fewer than 10 studies, funnel plot analysis was not performed.

#### 3.3.7. Longitudinal Trajectories of Key Biomarkers

**Figure 2:**
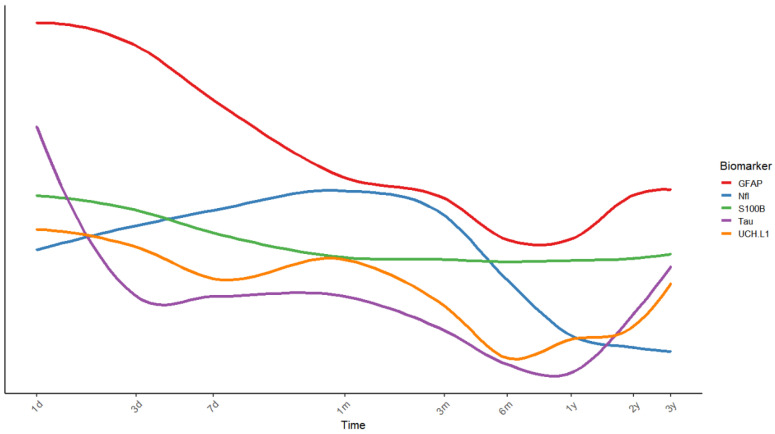
Longitudinal trajectories of biomarkers associated with TBI-CI. The smooth curve in the figure represents quantitatively derived data based on two studies with complete longitudinal sampling [30,46], with values log-transformed and plotted using R (R version 4.4.1). TBI: traumatic brain injury; CI: cognitive impairment.

Several studies included in this review have characterized the temporal dynamics of biomarkers following TBI, including NfL, Tau, UCH-L1, GFAP, NSE, and S100B [23,30,36,37,45,46,51,53,56] (Figure 2). Overall, many injury-related biomarkers exhibit an early peak within 24 h post-injury, followed by a gradual decline. For example, serum NSE levels peak within the first few hours after injury and then decrease over time [37], while S100B shows a similar acute-phase peak (≤24 h) with subsequent decline [30,37]. UCH-L1 typically peaks within 24–72 h post-injury, with limited evidence suggesting possible re-elevation in the chronic phase of sTBI [30,46,56]. Inflammatory markers such as IL-1*β*, IL-6, and IL-10 are also elevated in the acute phase and gradually decrease thereafter [56]. In contrast, NfL demonstrates a delayed and prolonged temporal profile, with levels increasing over days to weeks and remaining elevated for weeks to months before gradually declining over subsequent years [30,46,56]. Importantly, elevated levels at later timepoints (3–6 months) have been associated with brain atrophy [46]. Tau shows an acute increase followed by a decline within the first week, although a secondary elevation has been reported in a subset of patients with sTBI between 6 and 12 months [30,46,56]. GFAP exhibits a more complex trajectory. It typically peaks within the first day post-injury and subsequently declines [30]. However, longitudinal studies have reported secondary increases at later timepoints [23,36,45,46], including elevations from 6 months to 3 years in patients with TBI-CI [36]. In addition, progressive increases over 2–8 years have been associated with reaction time (*β* = 0.20, 95% *CI*: 0.097–0.303) [23] and brain atrophy [45].

These distinct temporal patterns likely reflect differences in the underlying pathophysiological processes following TBI. Early-peaking biomarkers, such as NSE, S100B, and UCH-L1, primarily reflect acute neuronal and astroglial injury, whereas delayed and sustained elevations of NfL are indicative of axonal degeneration. Notably, the biphasic or prolonged profiles of Tau and GFAP suggest that these markers capture secondary injury cascades, including chronic neuroinflammation and reactive gliosis, which are increasingly implicated in the development of long-term TBI-CI. Furthermore, these biomarker kinetics are critically modulated by the functional integrity of the blood–brain barrier and the efficiency of glymphatic clearance, suggesting that peripheral levels provide a composite signal of structural damage, neuroinflammation, and impaired protein homeostasis, rather than reflecting an isolated pathological event.

## 4. Discussion

In this meta-analysis, we conducted a comprehensive and systematic search and screened relevant literature, ultimately including 29 studies. Our findings revealed a robust association between TBI-CI and a wide range of biomarkers. In fact, no single biomarker is universally optimal across all clinical scenarios. We highlight BDNF and related synaptic proteins as key indicators associated with TBI-CI. For early screening and prediction, NfL, GFAP, UCH-L1, NSE and S100B measured within 72 h post-injury demonstrated the substantial evidence. For long-term prognosis and the discrimination of TBI-CI, plasma NfL and GFAP offer potential value. Elevated levels of T-tau in biofluids may reflect axonal injury and neurodegenerative process in TBI-CI. However, evidence remains lacking regarding their ability to differentiate between specific subtypes of CI. In addition, several studies used AD diagnosis as an endpoint, providing further evidence for a potential etiological link between TBI and AD development. This review further provided substantial evidence that biomarkers linked to neuronal injury are significantly elevated in individuals with TBI-CI.

### 4.1. Mechanisms Underlying TBI-CI: Pathophysiology and Biomarkers

The pathogenesis of TBI-CI involves multiple interrelated mechanisms, including axonal injury, neuronal degeneration, glial activation, neuroinflammation, and amyloid pathology. These cascades interact dynamically rather than operating in isolation, contributing to large-scale network dysfunction that manifests clinically as impairments in executive function, memory, and processing speed.

Axonal damage represents the earliest pathological event following mechanical trauma, resulting from cytoskeletal disruption and axonal shearing [73], which triggers the release of biomarkers such as NfL, T-tau, P-tau, and αII-spectrin degradation products [32,74,75,76,77,78]. Among these, NfL has emerged as a sensitive indicator of axonal injury and has been associated with the development of TBI-CI and brain atrophy. Elevated T-tau and P-tau levels correlate with TBI-CI and gray matter loss [30,46]. Although tau levels may decline over time, persistent elevation during the chronic phase is associated with long-term cognitive impairment, potentially reflecting tau-related neurodegenerative processes. Additional neuronal injury markers, including SNTF [77,79], UCH-L1, and NSE [78,80], are widely used indicators of neuronal damage, particularly in the acute phase. In contrast, reduced BDNF levels may indicate impaired synaptic plasticity and compromised neural repair capacity [64,65], contributing to persistent cognitive deficits.

Glial reactivity and neuroinflammation represent critical contributors to the TBI-CI. Astrocytic injury leads to increased circulating GFAP and S100B levels, which correlate with brain atrophy and cognitive decline [23,30,46,81,82]. In the acute phase of TBI, the disruption of the blood–brain barrier further exacerbates astrocytic activation, resulting in the release of pro-inflammatory cytokines [83] that contribute to cognitive dysfunction [19]. Meanwhile, the microglial and macrophages remain persistently activated post-TBI [84], with elevated levels of inflammatory mediators such as IL-1*β*, TNF-α, IL-6, CCL2, and CRP observed during both acute and recovery stages [24,34,52,54,55,56]. Our findings corroborate prior evidence [72] that neuroinflammation and glial activation constitute a pivotal mechanistic link between TBI and subsequent CI.

Axonal injury may also disrupt amyloid precursor protein processing, promoting intraxonal Aβ accumulation and neurodegeneration [85,86]. However, associations between Aβ levels and TBI-CI remain inconsistent, likely due to heterogeneity in injury severity, disease stage, sampling methods, and study design [41,42,43,57]. These discrepancies may also reflect the clinical heterogeneity of TBI-CI, which does not uniformly progress to AD. Further longitudinal studies are needed to clarify Aβ dynamics following TBI.

Taken together, these interconnected mechanisms illustrate how acute mechanical injury evolves into chronic neurodegenerative cascades, ultimately culminating in CI, thereby underscoring the critical need for efficient biomarkers for TBI-CI.

### 4.2. Association Between TBI and AD

TBI is increasingly recognized as a significant risk factor for the development of AD [87], although the underlying mechanisms remain incompletely understood. Evidence suggests shared pathological pathways link TBI-CI to TBI-associated AD (TBI-AD). Tau pathology exhibits paradoxical features in TBI-AD. Notably, our analysis indicates that AD patients with a history of TBI exhibit significantly elevated CSF T-tau levels [32,33], whereas CSF Aβ42 concentration does not appear to be significantly influenced by prior TBI [32,33]. This divergent pattern provides supporting evidence for the interpretation that TBI may preferentially exacerbate tau-related neurodegenerative processes rather than amplifying amyloid-β deposition. Accordingly, tau may represent not only a biomarker but also a potential contributor linking TBI to accelerated neurodegeneration and cognitive decline in AD. For instance, plasma P-tau 217 has diagnostic value for early-onset or atypical dementia [88], and specific P-tau subtypes can help distinguish AD from other dementias [89]. Given the substantial pathological heterogeneity of TBI-CI, specific biomarker subtypes of P-tau may have potential utility for longitudinal monitoring. These markers may help identify patterns suggestive of chronic neurodegenerative processes associated with TBI, including AD or chronic traumatic encephalopathy.

Synaptic dysfunction is a shared feature between TBI and AD [32]. Synaptic proteins like NPTX2 and SNAP-25 are potential biomarkers. While the NPTX2 level is typically reduced in AD [90], it is elevated in the CSF of AD patients with prior TBI [32] and linked to attentional deficits, possibly reflecting TBI-specific synaptic changes. SNAP-25, elevated in AD and correlated with cognitive decline [91], shows further upregulation in AD patients with TBI history, suggesting a role in TBI-AD pathogenesis.

Importantly, given the substantial heterogeneity of cognitive dysfunction following TBI, these findings derived from AD-spectrum populations with a history of TBI should be interpreted with caution and may not be directly generalizable to all forms of TBI-CI.

In summary, tau pathology and synaptic dysfunction appear to represent key mechanisms linking TBI to AD, although additional pathways require further investigation.

### 4.3. Comparison with Existing Literature and Clinical Implications

Early studies identified Aβ, specific neurotransmitters, and S100B as key biomarkers for TBI-CI, but their scope was relatively narrow [22]. Later reviews broadened this view, highlighting chronic-phase inflammation, glial activation, and neurodegeneration and focusing on the pathophysiology of chronic TBI (>6 months). It emphasized alterations in biomarker levels during the chronic phase and their association with long-term complications post-TBI, such as functional impairment, psychological symptoms, and cognitive decline [92]. Our research confirms these mechanisms but introduces novel frameworks linking acute biomarkers to chronic outcomes. Methodologically, our study distinguishes itself by: (1) focusing specifically on TBI-CI; (2) a longitudinal design that tracks biomarker trajectories from the acute phase through to the chronic stage; (3) applying quantitative analyses to link biomarkers with cognition; and (4) employing a rigorous systematic review methodology.

For clinical applications, blood-based biomarkers offer a less invasive alternative to CSF. While no single marker is highly specific, multi-marker panels show strong predictive value. One study found that combining P-tau, NfL, GFAP, IL-6, and TNF-α effectively identifies patients with TBI-CI (AUC = 0.85) [24]. Another panel of serum 5-hydroxytryptamine and S100β also demonstrated predictive utility (AUC = 0.810) [49]. This supports the clinical use of multi-marker strategies for TBI-CI prediction and diagnosis.

### 4.4. Limitations

Several limitations warrant consideration. Many of the included studies were characterized by small sample sizes, cross-sectional designs, short follow-up durations, and heterogeneous cognitive assessments, which limit the robustness of the pooled analyses. Therefore, caution is warranted when interpreting causal relationships or clinical applicability. A lack of standardized diagnostic criteria for TBI-CI and insufficient control for confounders further contributed to moderate- to low-quality studies [37,47,50,51,54,55]. Generalizability across ethnicities and TBI etiologies remains uncertain, and genetic and epigenetic factors were rarely examined. Finally, most studies focused on AD patients with remote TBI, limiting differentiation between TBI-AD, non-AD dementias, and TBI without CI. Critically, substantial pre-analytical and analytical heterogeneity across studies may have influenced biomarker quantification and cross-study comparability. Variations in assay sensitivity (ELISA or Simoa), sample matrices, pre-centrifugation processing delays, collection tube types, hemolysis, and repeated freeze–thaw cycles were rarely controlled or reported. These methodological discrepancies, alongside unaccounted biological confounders like renal clearance, can significantly alter circulating biomarker concentrations and compromise reproducibility. This led to our cautious interpretation of the results. Lastly, given the potential for publication bias, our conclusions should be viewed as preliminary and interpreted judiciously alongside this constraint.

Future large-scale, longitudinal studies with harmonized diagnostic frameworks and repeated biomarker measurements are needed to clarify the mechanistic links between TBI and cognitive decline.

## 5. Conclusions

This study provides a comprehensive synthesis of fluid biomarkers associated with TBI-CI. Biomarkers such as NfL, GFAP, T-tau, and UCH-L1 showed consistent associations with CI and brain volume changes following TBI. This review further underscores the critical temporal dynamics of fluid biomarkers in TBI-CI, revealing that stage-specific biomarker profiles mirror distinct underlying pathophysiological processes. The findings highlight axonal injury, glial activation, neuroinflammation, and synaptic dysfunction as central pathological mechanisms underlying TBI-CI. TBI-AD may be more closely linked to tau-related neurodegenerative processes. While no single biomarker is sufficient across all clinical contexts, combined assessment of multiple biomarkers may improve risk stratification and enhance predictive performance for cognitive outcomes. Future research should focus on establishing standardized diagnostic frameworks and prioritizing multimodal integration of fluid biomarkers and neuroimaging in large-scale longitudinal cohorts to improve the prediction and early identification of individuals at risk for TBI-CI and TBI-AD.

## Figures and Tables

**Table 1:** Characteristics of included studies.

Study	Country	Study Design	TBI Severity	Phase	Sample Size	Sample Type	Assay Method	Biomarkers	Post-TBI Biomarker Sampling Time	Assessment of CI	Time Point of Assessment	NOS
De Boussard CN 2005 [47]	Sweden	Cohort	Mild	Acute	TBI: 97	Serum	ELISA	S100B, S100A1B	24 h	Neuropsychological tests	3 m	4
Shahim P 2024 [25]	USA	Cohort	Mild, moderate, severe	Chronic	TBI: 143 HC: 39	Serum	Simoa	NfL, GFAP, T-tau, UCH-L1	0.7 y	Neuropsychological tests	1 m, 3 m, 6 m, 1 y, 2 y, 3 y, 4 y, 5 y	7
Christian LoBue2024 [48]	USA	Case–control	/	Chronic	TBI+: 101 TBI−: 303	Serum	Simoa	NfL, GFAP, T-tau, UCH-L1	3.55 y	MMSE	/	8
Slavoaca D 2020 [37]	Romania	Cohort	Moderate, severe	Acute	TBI: 62	Serum	ECLIA	NSE, S100	4 h, 72 h	MMSE, WAIS, VST	3 m	4
Jin G 2023 [49]	China	Case–control	/	/	TBI CI: 64 TBI no-CI: 58	Serum	ELISA	S100B	/	MoCA	/	6
Milleville KA 2021 [35]	USA	Cohort	Moderate, severe	Chronic	TBI: 139	Serum	Luminex™	IL-1*β*, IL-7, TNF-*α*, sIL-4R, sIL-6R, MIP-1b, RANTES, IL-10, sICAM1	1–3 m	Neuropsychological tests	6 m, 12 m	6
Dzyak L 2023 [36]	Ukraine	Cohort	Severe	/	TBI: 310	Serum	ELISA	GFAP, NCAM	/	Neuropsychological tests	6 m, 1 y, 3 y	5
Peltz CB 2020 [24]	USA	Cross-sectional	/	Chronic	TBI: 65 No TBI: 90	Plasma exosomes	Simoa	IL-6, NfL, TNF-α, GFAP, P-tau, T-tau, *α*-syn, Aβ42, IL-10	>2 y	MMSE, AVLT, WAIS-R	/	6
Suzan van Amerongen2024 [32]	Netherlands	Case–control	/	Chronic	AD TBI+: 110 AD TBI−: 110	CSF	ELISA	Aβ42, P-tau 181, T-tau, NfL, SNAP 25, Ng, NPTX2, GluR4	Baseline	Neuropsychological tests	2.2~8 y	7
Christian LoBue 2023 [33]	USA	Case–control	/	Chronic	AD TBI+: 10 AD TBI−: 20	CSF	Luminex™	Aβ42, P-tau 181, T-tau	Baseline	Neuropsychological tests	/	7
Lippa SM 2022 [40]	USA	Case–control	Mild, severe	/	TBI: 110 IC: 37 HC: 77	Serum	Simoa	T-tau, NfL, GFAP, UCH-L1	Within 1 y	Neuropsychological Assessment	/	6
Robert Siman 2013 [50]	USA	Cohort	Mild	Acute	TBI: 17 IC: 13 HC: 8	Serum	ECLIA	SNTF	~24 h	SDMT, KT	1 m, 3 m	5
Bernick C 2023 [23]	USA	Cohort	/	Chronic	Repetitive Head injury: 420 HC: 52	Plasma	Simoa	NfL, GFAP, P-tau 231, NTA	Baseline, 2 y, 4 y, 6 y, 8 y	CNS Vital Signs	2–3 y	6
Shahim P 2020 [46]	USA	Cohort	Mild, moderate, severe	Chronic	TBI: 162 HC: 68	Serum	Simoa	NfL, GFAP, UCH-L1, T-tau	30 d, 3 m, 6 m, 1 y, 2 y, 3 y, 4 y, 5 y	Brain volumes via MRI	6 m, 1 y, 2 y, 3 y, 4 y, 5 y	7
Ni P 2020 [51]	China	Cohort	Mild, moderate, severe	Acute	TBI: 229 HC: 30	Serum	ELISA	T-tau	1 d, 3 d, 5 d, 7 d, 14 d	MoCA	6 m	5
Neil Graham 2021 [30]	UK	Cohort	Moderate, severe	Subacute	TBI: 197 IC: 25	Plasma and serum	Simoa, ELISA	NfL, GFAP, UCH-L1, T-tau, S100B	~10 d, 10 d–6 w, 6 m, 12 m	Brain volumes via MRI	6 m, 1 y	7
Neil Graham 2023 [31]	UK	Cohort	Moderate, severe	Subacute	TBI: 42	Plasma	Simoa	P-tau 181	~10 d, 10 d–6 w, 6 m, 12 m	Brain volumes via MRI	6 m, 1 y	7
Asken BM 2023 [39]	USA	Cross-sectional	/	Chronic	Repetitive head injury: 33 HC: 59 AD: 62	Plasma	Simoa, CLIA	GFAP, NfL, IL-6, YKL40, IFN-γ	/	Neuropsychological tests, MRI	/	6
Sun Y 2019 [52]	China	Cohort	Mild	Acute	TBI: 95 HC: 54	Serum	Luminex™	CCL2, IL-1*β*	7 d	Neuropsychological tests	3 m	7
Alexandra L. Clark2021 [44]	USA	Cohort	/	Chronic	TBI: 52 HC: 50	CSF	ECLIA	P-tau, T-tau, Aβ42	/	Neuropsychological tests	/	7
Subir Dey 2017 [53]	India	Cohort	Mild	Acute	TBI: 20 HC: 20	Serum	ELISA	S100B, UCH-L1	6 h, 6 h~12 h	Neuropsychological tests	3 m	6
Newcombe VFJ 2022 [45]	UK	Cohort	Mild, moderate, severe	Chronic	TBI: 211 HC: 35	Serum	Simoa	GFAP, NfL	8 m, 5 y	Brain volumes via MRI	>5 y	8
Su SH 2013 [34]	China	Cohort	Mild	Subacute, chronic	TBI: 213	Plasma	LEIA	CRP	Baseline, 1 m, 2 m, 3 m	MoCA	3 m	7
Samatra DPGP 2018 [54]	Indonesia	Cohort	Mild	Acute	TBI: 70	Serum	ELISA	IL-1*β*	24 h	MoCA	7 d	5
Eagle SR 2024 [55]	USA	Cohort	Mild	Acute	TBI: 103	Plasma	ELISA	IL-1*β*, IL-18, Caspase-1	~24 h	TMT-A and B, WAIS	6 m, 12 m	6
Li G 2024 [41]	USA	Case–control	Mild	Chronic	TBI+: 51 TBI−: 85	CSF	CLIA, ELISA	T-tau, P-tau 181, Aβ42, Aβ40	/	Neuropsychological tests	/	7
Jia X 2023 [56]	China	Cohort	Mild	Acute	TBI: 103 HC: 66	Serum	Luminex™, Simoa	NfL, UCH-L1, IL-6, IL-1*β*, IL-10	7 d	Neuropsychological tests	1 m, 3 m, 6 m~1 y	7
Erica Howard 2024 [42]	USA	Cross-sectional	Mild, moderate, severe	Chronic	TBI: 56 HC: 56	CSF	MS/MS	Aβ42, Aβ40, Aβ38	44 y	Neuropsychological tests	/	7
Goetzl EJ 2020 [43]	USA	Cross-sectional	Mild, moderate, severe	Chronic	TBI: 47 No TBI: 61	Plasma exosomes	ELISA	P-tau 181, P-tau 396, Aβ42, IL-6, synaptogyrin-3 and PrPc	12–74 y	MMSE, AVLT, WAIS	/	6
Failla MD 2016 [38]	USA	Cohort	Severe	Acute	TBI: 113	Serum and CSF	ELISA	BDNF	0 d~6 d, 6 m, 12 m	FIM-Cog	6 m, 12 m	4

Notes: TBI: traumatic brain injury; d: day; y: year; m: month; HC: Healthy controls; IC: Injury controls not related brain; AD: Alzheimer’s disease; CSF: Cerebrospinal fluid; S100: S100 calcium binding protein; NfL: Neurofilament light chain; GFAP: Glial fibrillary acidic protein; T-tau: Total tau; P-tau: phosphorylation tau; UCH-L1: Ubiquitin C-terminal hydrolase-L1; NSE: neuron-specific enolase; α-syn: α-synuclein; Aβ: amyloid β-protein; PrPc: cellular prion protein; IL-1*β*: Interleukin-1*β*; IL-7: Interleukin-7; IL-10: Interleukin-10; IL-18: Interleukin-18; IL-6: Interleukin-6; TNF-*α*: Tumor Necrosis Factor *α*; sIL-4R: soluble IL-4 receptor; sIL-6R: soluble IL-6 receptor; MIP-1b: Macrophage Inflammatory Protein 1 β; RANTES: Regulated upon Activation, Normal T-cell Expressed and Secreted; sICAM1: soluble Intracellular Adhesion Molecule 1; SNAP 25: synaptosomal-associated protein 25 kDa; Ng: neurogranin; NPTX2: neuronal pentraxin 2; GluR4: glutamate receptor 4; SNTF: αII-Spectrin N-terminal fragment; CCL2: monocyte chemoattractant protein1; CRP: C-reactive protein; Caspase-1: cysteinyl aspartate specific proteinase 1; NCAM: neural cell adhesion molecule; BDNF: brain-derived neurotrophic factor; NTA: N-terminal tau; ELISA: enzyme-linked immunosorbent assay; TMT-A and B: Trail Making Test Part A and B; WAIS: the Wechsler Adult Intelligence Scale; MMSE: Mini-Mental State Examination; MoCA: Montreal Cognitive Assessment; AVLT: Auditory Verbal Learning Task; SDMT: Symbol-Digit Modalities Test; KT: Keep Track task; CNS Vital Signs: including verbal memory, symbol digit coding, Stoop, and a finger tapping test; FIM-Cog: Functional Independence Measure–Cognition; CLIA: chemiluminescence-based immunoassay; ECLIA: electrochemi-luminescence immunoassays; LEIA: latex-enhanced immunonephelometric assay; Simoa: Single Molecular Array; MS: mass spectrometry.

**Table 2:** Subgroup analysis of biomarkers.

TBI Type	Phase	N	Related Biomarkers	Conclusion
mTBI	Acute	7	NfL, UCH-L1, S100A1B, S100B, SNTF, CCL2, IL-1*β*, IL-18, Caspase-1, IL-6	Serum S100B and UCH-L1 measured within 24 h post-injury were associated with long-term cognitive function [53], as well as SNTF [50]. Additionally, serum NfL and UCH-L1 levels may predict subsequent brain atrophy and CI [56]. However, one study reported no significant association between S100A12 or S100B levels measured within 24 h and CI at 3 months [47]. Acute-phase IL-1*β* levels in serum and plasma predicted CI [52,54,55,56]. Similarly, serum IL-6 and CCL2 levels measured within 3 days post-injury may have a predictive ability of CI [52,56].
	Subacute	1	CRP	High plasma CRP levels at subacute stage were associated with a higher risk of persistent CI post-injury [34].
	Chronic	1	T-tau, P-tau 181, Aβ42, Aβ40	Lower CSF Aβ42 and Aβ40 levels were associated with CI over 45 years of age, while neither CSF P-tau181 nor T-tau level were correlated with cognitive performance [41].
sTBI	Acute	2	NSE, S100, BDNF	Serum BDNF [38] levels at acute stage associations with memory recovery. Acute-phase serum NSE levels may predict CI [37].
	Subacute	1	NfL, GFAP, UCHL1, T-tau, S100B, P-tau 181	The levels of plasma NfL, GFAP, UCH-L1, T-tau, serum S100B could predict brain atrophy [30,31].
	Chronic	1	IL-1β, IL-7, TNF α, sIL-4R, sIL-6R, MIP-1b, RANTES, IL-10, sICAM1	All of those biomarkers in serum could predict long-term cognitive dysfunction except IL-1*β* [35].
	All stage	1	GFAP, NCAM	Elevated serum GFAP and NCAM levels may serve as useful biomarkers for differentiating between TBI patients with and without CI [36].
All TBI	Acute	1	T-tau	Serum T-tau levels during the acute phase may serve as a potential biological marker for the early diagnosis and assessment of TBI-CI [51].
	Chronic	5	NfL, GFAP, T-tau, UCH-L1, Aβ42, Aβ40, Aβ38, P-tau 181, P-tau 396, IL-6, synaptogyrin-3 and PrPc	The levels of serum NfL, GFAP, T-tau, UCH-L1 [25,45,46], as well as CSF Aβ40 [42], have been shown to correlate with cognitive function following TBI. Additionally, levels of plasma exosomes P-tau 181, P-tau 396, Aβ42, PrPc, and synaptogyrin-3 were significantly elevated in individuals with CI compared to those without CI, several years post-TBI [43].
All TBI	/	1	T-tau, NfL, GFAP, UCH-L1	These biomarkers in serum could be helpful in predicting those at risk for TBI-CI [40].
Repetitive TBI	Chronic	2	NfL, GFAP, P-tau 231, NTA, IL-6	Only plasma NfL and GFAP were associated with cognitive performance [23,39]. Elevated concentrations of these biomarkers may aid in the identification of brain atrophy and CI after repetitive TBI [23].

Notes: TBI: traumatic brain injury; CI: cognitive impairment; CSF: Cerebrospinal fluid; S100: S100 calcium binding protein; NfL: neurofilament light chain; GFAP: glial fibrillary acidic protein; T-tau: total tau; P-tau: phosphorylation tau; UCH-L1: Ubiquitin C-terminal hydrolase-L1; NSE: neuron-specific enolase; Aβ: amyloid β-protein; PrPc: cellular prion protein; IL-1*β*: Interleukin-1 *β*; IL-7: Interleukin-7; IL-10: Interleukin-10; IL-18: Interleukin-18; IL-6: Interleukin-6; TNF *α*: Tumor Necrosis Factor *α*; sIL-4R: soluble IL-4 receptor; sIL-6R: soluble IL-6 receptor; MIP-1b: Macrophage Inflammatory Protein 1 β; RANTES: Regulated upon Activation, Normal T-cell Expressed and Secreted; sICAM1: soluble Intracellular Adhesion Molecule 1; SNTF: αII-Spectrin N-terminal fragment; CCL2: monocyte chemoattractant protein1; CRP: C-reactive protein; Caspase-1: cysteinyl aspartate specific proteinase 1; NCAM: neural cell adhesion molecule; BDNF: brain-derived neurotrophic factor.

**Table 3:** The distribution of biomarkers from different body fluid sources.

Biomarkers	Fluid Types	Results
T-tau	CSF	Compared with AD patients without a history of TBI, AD patients with a history of TBI showed significantly higher levels of T-tau in cerebrospinal fluid [32,33].
	Serum/Plasma	The level of serum or plasma T-tau may predict or identify TBI-CI [30,46,51].
P-tau	CSF	Although general P-tau elevation associated with TBI-CI [44], the specific P-tau 181 biomarker is neither driven by TBI history in AD [32,33] nor associated with cognitive or volumetric outcomes following TBI [31,32,41].
	Plasma exosomes	Elevated levels of P-tau in plasma exosomes, including higher concentrations of P-tau 181 and P-tau 396, could distinguish veterans with TBI-CI and associated with TBI-CI [24,43].
Aβ42	CSF	In AD, a history of TBI is not a determinant of cerebrospinal fluid Aβ42 levels [32,33]. Conversely, within TBI cohorts, low CSF Aβ42 serves as an indicator of poor prognosis, identifying patients with worse cognitive function [41].
	Plasma exosomes	While elevated plasma exosomal Aβ42 levels were observed in TBI-CI [43], other studies did not show a significant difference [24], indicating the need for further investigation.
BDNF	CSF	CSF BDNF levels did not correlate significantly with cognitive outcomes post-TBI [38].
	Serum	The acute-phase level correlated with TBI-CI [38].

Notes: TBI: traumatic brain injury; CI: cognitive impairment; TBI-CI: TBI-related CI; T-tau: total tau; P-tau: phosphorylation tau; Aβ: amyloid β-protein; CSF: Cerebrospinal fluid; AD: Alzheimer’s disease; BDNF: brain-derived neurotrophic factor.

## Data Availability

No new data were created or analyzed in this study. Data sharing is not applicable to this article.

## Supplementary Materials

The following supporting information can be downloaded at: https://www.mdpi.com/article/10.3390/ijms27104274/s1.
