# Supplementary material for: Fluid Biomarkers of Cognitive Impairments Following Traumatic Brain Injury: A Systematic Review and Meta Analysis
Source: Int J Mol Sci. 2026 May 11;27(10):4274. doi: 10.3390/ijms27104274 (PMC13207309; doi:10.3390/ijms27104274)
Supplement: Supplementary file 1 [file ijms-27-04274-s001.zip › ijms-4231670-supplementary.pdf]

Search strategy in database

**Pubmed**	
#1	("Brain Injuries, Traumatic"[Mesh]) OR (brain injury*[All Fields]) OR (TBI[All Fields]) OR (Traumatic Brain Injury[All Fields]) OR (Brain Trauma[All Fields]) OR (Traumatic Encephalopathy[All Fields]) OR (Traumatic Encephalopathies[All Fields]) OR (“Traumas, Brain”[All Fields]) OR (head injury[All Fields])
#2	"Cognitive Dysfunction"[Mesh] OR "Alzheimer Disease"[Mesh] OR (Cognitive impairment*[All Fields]) OR (cognitive disorder*[All Fields]) OR (Mental Deterioration[All Fields]) OR (Dementia[All Fields]) OR (cognitive decline[All Fields]) OR (neurodegeneration[All Fields]) OR (brain atrophy[All Fields])
#3	(biomarker[Title/Abstract]) OR (evaluation[Title/Abstract]) OR (assessment[Title/Abstract]) OR (biomarkers[Title/Abstract]) OR (relationship[Title/Abstract]) OR (marker[Title/Abstract])
#4	#1 and #2 and #3
**Embase**	
#1	('traumatic brain injury'/exp) OR (“brain injury”:ti,ab) OR (TBI:ti,ab) OR (“Brain Trauma”:ti,ab) OR (“Traumatic Encephalopathy”:ti,ab) OR (“Traumatic Encephalopathies”:ti,ab) OR (“head injury”:ti,ab)
#2	'dementia'/exp OR 'mild cognitive impairment'/exp OR ("Cognitive Dysfunction":ti,ab) OR (“Cognitive impairment”:ti,ab) OR (“cognitive disorder”:ti,ab) OR (“Mental Deterioration”:ti,ab) OR (“cognitive decline”:ti,ab) OR (“neurodegeneration”:ti,ab) OR (“brain atrophy”:ti,ab)
#3	biomarker:ti,ab OR evaluation:ti,ab OR assessment:ti,ab OR risk:ti,ab OR biomarkers:ti,ab OR relationship:ti,ab OR association:ti,ab
#4	#1 and #2 and #3
**Cochrane library**	
#1	(Brain injuries[mh])
#2	(Cognitive Dysfunction[mh]) OR ((cognitive decline) OR (neurodegeneration) OR (brain atrophy) OR (Dementia) OR (Alzheimer Disease)):ti,ab,kw
#3	(biomarker OR evaluation OR assessment OR risk OR biomarkers OR relationship OR association):ti,ab,kw
#4	#1 AND #2 AND #3

Supplementary Table S1 The association with neuronal biomarkers and cognition post-TBI

Study	Measure time	Statistical method	Adjust covariates	N	Cognitive function(time)	Correlation or regression coefficient		*P* or 95%*CI*
NfL[1]	/	Pearson	Age，sex	33	Memory	r	-0.24	/
NfL[2]	~6w	Linear regression models	/	140	Lower volumes of total GM at 1m~6m	R^2	**0.20**	**0.001**
NfL[3]	1y	Linear regression models	/	23	Perceptual Reasoning Index over time	R^2	**0.211**	**0.012**
NfL[4]	Baseline	Linear regression models	Age, race,sex, education, and time	140	Volumes of the thalamus	β	**-1.68**	**[-2.77, -0.61]**
				140	Volumes of the hippocampus	β	**-0.84**	**[-1.56, -0.112]**
				140	Volumes of the subcortical gray matter	β	**-7.9**	**[-14.3, -1.47]**
NfL[5]	30d	Spearman	/	162	Lower volumes of total GM	ρ	**-0.41**	**＜0.0001**
				162	Lower volumes of total WM	ρ	**-0.31**	**0.00036**
	6m	Linear regression models	Age, education, and sex	/	Lower volumes of total WM at 1y	β	**-3881**	**0.001**
NfL[6]	~8m	Linear regression models	Age, sex, duration of follow-up	/	Lower volumes of total WM at >5y	R^2	**0.41**	**0.04**
NfL[7]	/	Linear mixed models	Education	220	MMSE over time	β	0.055	0.147
					Attention over time	β	-0.036	0.516
					Executive function over time	β	0.023	0.651
					Language over time	β	0.031	0.574
					Memory over time	β	0.05	0.253
NfL[8]	0.7y	Linear models	Age, sex, education and time	143	Executive function at baseline	β	**-0.22**	**＜0.05**
	Longitudinal changes	Spearman	/	82	Longitudinal changes in whole brain WM volume	ρ	**0.33**	**0.013**
				71	Longitudinal changes in attention	ρ	-0.10	0.71
				71	Longitudinal changes in executive function	ρ	-0.11	0.74
				71	Longitudinal changes in language	ρ	-0.11	0.80
				71	Longitudinal changes in delayed	ρ	-0.32	0.09

					memory			
				71	Longitudinal changes in working memory	ρ	-0.08	0.72
NfL[9]	2.84d	Linear regression models	Age	103	The Wechsler Adult Intelligence Scale III Digital Symbol Coding	β	0.152	0.403
				77	WM atrophy(3m)	β	-0.149	0.491
				77	WM atrophy(>3m)	β	**-0.589**	**0.019**
				77	GM atrophy (3m)	β	-0.291	0.185
				77	GM atrophy(>3m)	β	**-0.571**	**0.023**
T tau[7]	/	Linear mixed models	Education	220	MMSE over time	β	0.039	0.326
					Attention over time	β	-0.049	0.371
					Executive function over time	β	-0.028	0.565
					Language over time	β	0.038	0.492
					Memory over time	β	0.028	0.545
T tau [8]	Longitudinal changes	Spearman	/	82	Longitudinal changes in whole brain WM volume	ρ	0.18	0.11
				70	Longitudinal changes in attention	ρ	0.02	0.97
				70	Longitudinal changes in executive function	ρ	0.06	0.79
				70	Longitudinal changes in language	ρ	-0.04	0.92
				70	Longitudinal changes in delayed memory	ρ	-0.12	0.89
				71	Longitudinal changes in working memory	ρ	-0.27	0.20
T tau [3]	1y	Spearman	/	55	Perceptual Reasoning Index at baseline	r	0.458	**0.049**
					Immediate Memory Index	r	0.532	**0.009**

					Delayed Memory Index	r	0.449	**0.032**
		Linear regression models	Age, education, sex,race,injury	23	Perceptual Reasoning Index over time	R^2	0.188	**0.033**
			severity	23	Executive function over time	R^2	0.244	**0.007**
T tau [5]	30d	Spearman	/	162	Lower volumes of total GM	ρ	-0.27	**0.0022**
				162	Lower volumes of total WM	ρ	0.04	0.62
	3m	Linear regression models	Age, education, and sex	/	Lower volumes of total WM at 1y	β	-3408	0.06
T tau [10]	1d	Pearson	/	95	MoCA at 6m	r	0.148	**0.0014**
	3d				MoCA at 6m		0.0835	**0.0206**
	5d				MoCA at 6m		0.0913	**0.0389**
	7d				MoCA at 6m		0.0536	0.1372
	14d				MoCA at 6m		0.0259	0.6177
T tau [2]	~6w	Linear regression models	/	140	Lower volumes of total GM at 1m~6m	R^2	0.34	**0.001**
T tau [11]	Baseline	Linear regression models	Age, education, APOE4	52	Processing speed	β	4.2	0.06
			positivity		Executive function	β	8.21	0.08
					Verbal learning	/	/	>0.05
					Memory composites	/	/	>0.05
P tau 181[7]	/	Linear mixed models	Education	220	MMSE over time	β	0.023	0.568
					Attention over time	β	-0.081	0.145
					Executive function over time	β	-0.033	0.485
					Language over time	β	0.01	0.918
					Memory over time	β	0.009	0.838
P tau[11]	Baseline	Linear regression models	Age, education, APOE4	52	Processing speed	β	-3.74	**0.009**
			positivity		Executive function	β	-5.89	**0.05**
					Verbal learning	/	/	>0.05
					Memory composites	/	/	>0.05
UCHL-1[8]	0.7y	Spearman	/	143	Delayed memory composites over time	r	0.3	**0.017**

	Longitudinal changes	Spearman	/	82	Longitudinal changes in whole brain WM volume	ρ	-0.14	0.33
				70	Longitudinal changes in attention	ρ	-0.005	0.99
				70	Longitudinal changes in executive function	ρ	-0.003	0.98
				70	Longitudinal changes in language	ρ	-0.12	0.82
				70	Longitudinal changes in delayed memory	ρ	0.23	0.38
				71	Longitudinal changes in working memory	ρ	0.11	0.72
UCHL-1[9]	2.84d	Linear regression models	Age	103	The impairment of cognitive flexibility(>6m)	β	-0.597	**＜0.001**
				77	WM atrophy(3m)	β	0.116	0.481
				77	WM atrophy(>3m)	β	0.218	0.376
				77	GM atrophy (3m)	β	0.151	0.361
				77	GM atrophy(>3m)	β	0.298	0.206
UCHL-1[2]	~6w	Linear regression models	/	140	Lower volumes of total GM at 1m~6m	R^2	0.25	**<0.001**
UCHL-1[3]	1y	Spearman	/	32	Perceptual Reasoning Index at baseline	r	-0.485	**0.006**
		Spearman	/	27	Working Memory Index at baseline	r	0.355	**0.042**
UCHL-1[3]	1y Longitudinal changes	Linear regression models	Age, years of education, number of TBIs, number of blasts, number of deployments, sex, race, and time since injury	27	Slower processing speed at baseline	β	-0.395	**0.026**
				28	Longitudinal changes Immediate Memory Index	β	-0.476	**0.006**
				28	Longitudinal changes Delayed Memory Index	β	-0.633	**0.002**
UCHL-1[5]	30d	Spearman	/	162	Lower volumes of total GM at baseline	ρ	-0.27	**0.0024**
				162	Lower volumes of total WM at baseline	ρ	-0.15	**0.05**

UCHL-1[12]	3.7h	Spearman	/	20	Digit Span at 3m	ρ	0.126	>0.05
				20	Spatial span at 3m at 3m	ρ	0.359	>0.05
				20	COWA (average words) at 3m	ρ	0.019	>0.05
				16	Stroop Test at 3m	ρ	-0.132	>0.05
				18	Trail making 1 at 3m	ρ	-0.027	>0.05
				18	Trail making 2 at 3m	ρ	0.363	>0.05
				20	AVLT–learning at 3m	ρ	0.529	**0.05**
				20	AVLT–immediate recall at 3m	ρ	0.365	>0.05
				20	AVLT–delayed recall at 3m	ρ	0.278	>0.05
				17	CFT–copy at 3m	ρ	-0.164	>0.05
				17	CFT–immediate recall at 3m	ρ	−0.218	>0.05
				20	CFT–delayed recall at 3m	ρ	0.062	>0.05
UCHL-1[12]	8.65h	Spearman	/	20	Digit Span at 3m	ρ	0.185	>0.05
				20	Spatial span at 3m	ρ	0.65	**<0.01**
				20	COWA (average words) at 3m	ρ	-0.54	>0.05
				16	Stroop Test at 3m	ρ	0.191	>0.05
				18	Trail making 1 at 3m	ρ	-0.148	>0.05
				18	Trail making 2 at 3m	ρ	0.147	>0.05
				20	AVLT–learning at 3m	ρ	-0.335	>0.05
				20	AVLT–immediate recall at 3m	ρ	0.221	>0.05
				20	AVLT–delayed recall at 3m	ρ	0.064	>0.05
				17	CFT–copy at 3m	ρ	-0.469	**0.05**
				17	CFT–immediate recall at 3m	ρ	-0.090	>0.05
				20	CFT–delayed recall at 3m	ρ	0.009	>0.05
Aβ42[7]	/	Linear mixed models	Education	220	MMSE	β	-0.057	0.704
					Attention	β	-0.076	0.218

					Executive function	β	-0.057	0.291
					Language	β	-0.027	0.656
					Memory	β	0.054	0.271
Aβ42[13]	Baseline	Spearman	/	112	Cognitive performance	/	/	＞0.05
Aβ40[13]				112	Boston naming test performance	ρ	-0.2	**0.032**
Aβ38[13]				112	Boston naming test performance	ρ	-0.19	**0.048**
Aβ40[13]		Linear regression models	Test time	56	Boston naming test performance	β	-0.14	**0.046**
Aβ38[13]				56	Boston naming test performance	/	/	＞0.05
Aβ42[11]	Baseline	Linear regression models	Age, education, APOE4 positivity	52	Processing speed	β	0.0001	0.62
					Executive function	β	0.0001	0.72
					Verbal learning	/	/	>0.05
					Memory composites	/	/	>0.05
NSE[14]	4h	Standardized regression paths	/	62	Cognitive status at 10d	β	-0.388	**0.034**
					Cognitive status at 3m	β	-0.411	**0.023**
NSE[14]	72h	Standardized regression paths	/	62	Cognitive status at 10d	β	-0.052	0.792
					Cognitive status at 3m	β	-0.067	0.729
Serum BDNF[15]	~6d	Spearman	/	30	Memory composite scores at 6m	r	0.53	0.005
				26	Memory composite scores at 12m	r	0.38	0.018
				45	FIM-Cog at 6m	r	0.31	0.041
Serum BDNF[15]	6m,12m	Spearman	/	47	FIM-Cog at 6m	r	0.16	0.271
CSF BDNF[15]	~6d	Spearman	/	53	FIM-Cog at 6m	r	-0.24	0.09

Notes: TBI: traumatic brain injury; d:day; y: year; m: moth; h:hour; w:week; NfL: Neurofilament light chain; GFAP: Glial fibrillary acidic protein; T tau: Total tau; P tau: phosphorylation tau; UCH-L1: Ubiquitin C-terminal hydrolase-L1; Aβ: amyloid β-protein; GM: grey matter; WM: white matter; MMSE: Mini-Mental State Examination; MoCA: Montreal Cognitive Assessment; AVLT: Auditory Verbal Learning Task; COWA: controlled oral word association tests; CFT: complex figure test; CI: confidence interval； BDNF: brain derived neurotrophic factor.

Supplementary Table S2 The association with other biomarkers and cognition post-TBI

Study	Measure time	Statistical method	Adjust covariates	N	Cognitive function(time)	Correlation or regression coefficient		P
SANP25[7]	Baseline	Linear mixed models	Years of education	220	MMSE over time	β	0.041	0.323
					Attention over time	β	-0.061	0.306
					Executive function over time	β	-0.038	0.459
					Language over time	β	0.045	0.440
					Memory over time	β	-0.013	0.769
NPTX2[7]	Baseline	Linear mixed models	Years of education	220	MMSE over time	β	0.017	0.659
					Attention over time	β	-0.116	**0.045**
					Executive function over time	β	-0.019	0.715
					Language over time	β	-0.009	0.867
					Memory over time	β	-0.012	0.780
GluR4[7]	Baseline	Linear mixed models	Years of education	220	MMSE over time	β	0.03	0.479
					Attention over time	β	-0.088	0.203
					Executive function over time	β	-0.007	0.908
					Language over time	β	0.008	0.913
					Memory over time	β	0.013	0.793
Ng[7]	Baseline	Linear mixed model	Years of education	220	MMSE over time	β	0.043	0.297
					Attention over time	β	-0.028	0.604
					Executive function over time	β	-0.021	0.662
					Language over time	β	0.069	0.187
					Memory over time	β	-0.014	0.741
Serum	~6d	Spearman	/	30	Memory composite scores at 6m	r	0.53	0.005

BDNF[15]				26	Memory composite scores at 12m	r	0.38	0.018
				45	FIM-Cog at 6m	r	0.31	0.041
Serum BDNF[15]	6m,12m	Spearman	/	47	FIM-Cog at 6m	r	0.16	0.271
CSF BDNF[15]	~6d	Spearman	/	53	FIM-Cog at 6m	r	-0.24	0.09

Notes: TBI: traumatic brain injury; d: day; ACTH: adrenocorticotropic hormone; TC: total cholesterol; CSF: Cerebrospinal fluid；CI: confidence interval；SNAP 25: synaptosomal-associated protein 25kDa; Ng: neurogranin; NPTX2: neuronal pentraxin 2; GluR4: glutamate receptor 4;

Supplementary Table S3 The association with Astrogliosis biomarkers and cognition post-TBI

Study	Measure time	Statistical method	Adjust covariates	N	Cognitive function(time)	Correlation or regression coefficient		P or 95%CI
GFAP[8]	0.7y	Linear models	Age, sex, education, and time since most recent injury	143	Executive function at baseline	β	-0.19	**0.05**
GFAP[8]	Longitudinal changes	Spearman	/	82	Longitudinal changes in whole brain WM volume	ρ	-0.03	0.21
				71	Longitudinal changes in attention	ρ	0.10	0.70
				71	Longitudinal changes in executive function	ρ	0.04	0.91
				71	Longitudinal changes in language	ρ	-0.02	0.96
				71	Longitudinal changes in delayed memory	ρ	-0.14	0.83
				72	Longitudinal changes in working memory	ρ	-0.12	0.94
GFAP[3]	Baseline	Spearman	/	55	Perceptual Reasoning Index at baseline	r	-0.333	**0.021**
		Linear regression models	Age,education, sex,race,injury severity	55	Slower processing speed at baseline	β	-0.378	**0.008**
GFAP[4]	Baseline	Linear regression	Age, gender, race, education years, and number of fights	140(mean age,31.03)	Lower volumes of the thalamus at baseline	β	-2.46	**[-4.00, -0.93]**
					Lower volumes of the hippocampus at baseline	β	-0.2	[-1.23, 0.84]

		models			Lower volumes of the subcortical gray matter at baseline	β	-7.62	[-16.8, 1.57]
GFAP[4]	Baseline	Linear regression models	Age, gender, race, education years, and number of fights	69(mean age,48.75)	Lower volumes of the thalamus at baseline	β	-4.12	**[-6.44, -1.81]**
					Lower volumes of the hippocampus at baseline	β	-1.9	**[-3.46, -0.34]**
					Lower volumes of the cerebral white matter at baseline	β	-40.72	**[-70.4,- 11.1]**
					Lower volumes of the total gray matter at baseline	β	-7.92	**[-11.1, -3.92]**
					Processing speed at baseline	β	-0.25	**[-0.38, -0.11]**
					Longitudinal rate of change in the thalamus	β	-4.64	**[-6.74, -2.54]**
					Longitudinal rate of change in the hippocampus	β	-2.52	**[-3.86, -1.18]**
					Longitudinal change of processing speed	β	-381	**[-552, -210]**
					Longitudinal change of reaction time	β	0.77	[0.31, 1.23]
GFAP[4]	Longitudinal GFAP levels	Linear regression models	Age, gender, race, education years, and number of fights	69(mean age,48.75)	Longitudinal change of reaction time	β	0.2	**[0.097, 0.303]**
GFAP[5]	30d	Spearman	/	162	Lower volumes of total GM at baseline	ρ	-0.39	**＜0.0001**
				162	Lower volumes of total WM at baseline	ρ	-0.34	**＜0.0001**
GFAP[2]	~6w	Linear regression models	/	140	Lower volumes of total GM at 1m~6m	R^2	0.27	**<0.001**
GFAP[1]	/	Pearson	Age, sex	33	Memory	r	-0.51	/
GFAP[6]	8m	Linear regression models	Age, sex，duration of follow-up	/	Total WM injury at >5y	R^2	0.27	0.171
S100B[16]	/	/	/	64	MoCA	r	-0.436	**＜0.05**
S100B[2]	6w	Linear	/		Atrophy rate of gray matter at 6m	Adj R^2	0.21	**0.004**

		regression						
S100B[12]	3.7h	Spearman	/	20	Digit Span at 3m	ρ	−0.481	**0.05**
				20	Spatial span at 3m	ρ	0.026	>0.05
				20	COWA (average words) at 3m	ρ	−0.342	>0.05
				16	Stroop Test at 3m	ρ	−0.099	>0.05
				18	Trail making 1 at 3m	ρ	−0.002	>0.05
				18	Trail making 2 at 3m	ρ	0.305	>0.05
				20	AVLT–learning at 3m	ρ	−0.559	**0.01**
				20	AVLT-immediate recall at 3m	ρ	0.286	>0.05
				20	AVLT-delayed recall at 3m	ρ	0.291	>0.05
				17	CFT-copy at 3m	ρ	0.129	>0.05
				17	CFT-immediate recall at 3m	ρ	0.083	>0.05
				20	CFT-delayed recall at 3m	ρ	0.188	>0.05
S100B[12]	8.65h	Spearman	/	20	Digit Span at 3m	ρ	−0.457	**0.05**
				20	Spatial span at 3m	ρ	0.195	>0.05
				20	COWA (average words) at 3m	ρ	−0.508	**0.05**
				16	Stroop Test at 3m	ρ	−0.049	>0.05
				18	Trail making 1 at 3m	ρ	−0.105	>0.05
				18	Trail making 2 at 3m	ρ	0.6	>0.05
				20	AVLT-learning at 3m	ρ	−0.602	**0.01**
				20	AVLT-immediate recall at 3m	ρ	0.331	>0.05
				20	AVLT-delayed recall at 3m	ρ	0.354	>0.05
				17	CFT-copy at 3m	ρ	0.235	>0.05
				17	CFT–immediate recall at 3m	ρ	0.26	>0.05
				20	CFT–delayed recall at 3m	ρ	0.39	>0.05
S100[14]	4h	SEM	/	62	Cognitive status at 10d	β	0.072	0.66

				62	Cognitive status at 90d	β	0.136	0.396
	72h			62	Cognitive status at 10d	β	-0.095	0.515
				62	Cognitive status at 90d	β	-0.213	0.138

Notes: TBI: traumatic brain injury; d: day; y: year; m: moth; h: hour; w: week; S100: S100 calcium-binding protein; SEM: Structural Equation Modeling; GM: grey matter; WM: white matter; MoCA: Montreal Cognitive Assessment; AVLT: Auditory Verbal Learning Task; COWA: controlled oral word association tests; CFT: complex figure test; CI: confidence interval

Supplementary Table S4 The association with inflammatory biomarkers and cognition post-TBI

Markers	Measure time	Statistical method	Adjust covariates	N	Cognitive function(time)	Correlation or regression coefficient		P
IL-1β[9]	2.84d	Linear regression models	Age	103	The impairment of cognitive flexibility(>6m)	β	-0.412	**0.017**
				77	WM Atrophy (<3m)	β	-0.009	0.954
				77	WM Atrophy (3m~1y)	β	0.154	0.548
				77	GM Atrophy (<3m)	β	0.027	0.872
				77	GM Atrophy (3m~1y)	β	0.227	0.371
IL-1β[17]	1m-3m	Linear regression models	/	119	Cognitive composite score at 6m	β	-1.54	0.066
				125	Cognitive composite score at 1y	β	1.35	0.077
				131	Memory at 6m	β	-2.11	0.06
				129	Memory at 1y	β	-1.87	0.077
				129	Attention & Processing Speed at 1y	β	-1.74	0.097
IL-1β[18]	24h	Chi-square test	/	70	Cognitive function at 7d	RR	2.6	**<0.001**
IL-1β[19]	<7d	Multiple Linear regression analysis	/	95	Working memory at baseline	β	-0.187	**0.047**
IL-1β[20]	1d	Multivariable Linear regression models	/	103	Trail Making Test Part A at 6m	β	-3.62	**0.01**
					Trail Making Test Part A at 1y	β	-4.15	**0.03**
					Trail Making Test Part B at 6m	β	-2.89	**0.06**
					Trail Making Test Part B at 1y	β	-3.66	**0.046**

CCL2[19]	2.63d	Multiple Linear regression analysis	/	52	Digital symbol coding test score at 3m	β	0.214	**0.009**
IL-6[9]	2.84d	Linear regression models	Age	103	The impairment of cognitive flexibility(>6m)	β	-0.392	**0.026**
IL-6[1]	/	Pearson	Age, sex	33	Memory	r	0.08	/
					Executive function	r	-0.02	/
					Language	r	-0.1	/
					Visuospatial	r	-0.05	/
CRP[21]	<1m	Multiple logistic regression models	Age, gender	213	MoCA at 3m	RR	1.657	**0.014**
	Baseline （<1m）	Multiple logistic regression models	Age, gender	213	Cognitive impairment	r	0.177	**0.01**
IL-7[17]	1m-3m	Linear regression models	/	119	Cognitive composite score at 6m	β	-1.48	0.09
				125	Cognitive composite score at 1y	β	-1.63	**0.042**
				124	Memory at 6m	β	-2.37	**0.04**
				131	Memory at 1y	β	-2.11	0.052
				129	Attention & Processing Speed at 1y	β	-1.8	0.099
TNF α[17]	1m-3m	Linear regression models	/	119	Cognitive composite score at 6m	β	-1.38	0.093
				125	Cognitive composite score at 1y	β	1.44	**0.055**
				131	Memory at 1y	β	1.87	0.073
sIL-4R[17]	1m-3m	Linear regression models	/	119	Cognitive composite score at 6m	β	2.34	**0.012**
				125	Cognitive composite score at 1y	β	1.54	0.051
				124	Memory at 6m	β	3.31	**0.007**
				126	Attention & Processing Speed at 6m	β	2.54	**0.026**
				131	Memory at 1y	β	2.78	**0.009**
				129	Attention & Processing Speed at 12M	β	2.30	**0.029**
				125	Executive function at 6m	β	2.30	**0.04**

sIL-6R[17]	1m-3m	Linear regression models	/	119	Cognitive composite score at 6m	β	-1.92	**0.036**
				125	Cognitive composite score at 1y	β	-1.45	0.077
				126	Attention & Processing Speed at 6m	β	-1.88	0.090
				123	Verbal fluency at 6m	β	-2.46	**0.013**
MIP-1b[17]	1m-3m	Linear regression models	/	119	Cognitive composite score at 6m	β	-1.84	**0.026**
				125	Cognitive composite score at 1y	β	-1.79	**0.017**
				124	Memory at 6m	β	-3.1	**0.005**
				131	Memory at 1y	β	-2.76	**0.008**
				129	Attention & Processing Speed at 1y	β	-2.18	**0.035**
RANTES[17]	1m-3m	Linear regression models	/	119	Cognitive composite score at 6m	β	-1.62	0.097
				125	Cognitive composite score at 1y	β	-2.42	**0.004**
				131	Memory at 1y	β	-2.58	**0.025**
				129	Attention & Processing Speed at 1y	β	-2.35	**0.042**
				129	Executive function at 1y	β	-1.81	**0.034**
IL-18[20]	1d	Multivariable Linear regression models	/	103	Trail Making Test Part A at 6m	β	-1.62	0.47
					Trail Making Test Part A at 1y	β	-1.1	0.6
					Trail Making Test Part B at 6m	β	0.66	0.78
					Trail Making Test Part B at 1y	β	-0.82	0.69
IL-10[17]	1m-3m	Linear regression models	/	124	Memory at 6m	β	2.78	**0.03**
IL-10[9]	1m	Linear regression models	Age	103	The impairment of cognitive flexibility at 1m	β	-0.106	0.302
sICAM1[17]	1m-3m	Linear regression models	/	123	Verbal fluency 6M	β	-2.46	**0.022**
				130	Verbal fluency 12M	β	-2.04	**0.04**
Caspase-1[20]	1d	Multivariable Linear regression models	/	103	Trail Making Test Part A at 6m	β	0.87	0.4
					Trail Making Test Part A at 1y	β	0.15	0.9
					Trail Making Test Part B at 6m	β	-0.29	0.79
					Trail Making Test Part B at 1y	β	-0.71	0.56

Notes: TBI: traumatic brain injury; d: day; y: year; m: moth; h: hour; w: week; IL-1β: Interleukin-1 β; IL-7: Interleukin-7; IL-10:Interleukin-10; IL-18: Interleukin-18; IL-6: Interleukin-6;TNFα: Tumor Necrosis Factor α; sIL-4R: soluble IL-4 receptor; sIL-6R: soluble IL-6 receptor; MIP-1b: Macrophage Inflammatory Protein 1 β; RANTES: Regulated upon Activation, Normal T-cell Expressed and Secreted; sICAM1：soluble Intracellular Adhesion Molecule 1; CCL2：monocyte chemoattractant protein1; CRP: C-reactive protein; Caspase-1: cysteinyl aspartate specific proteinase 1; CI: confidence interval

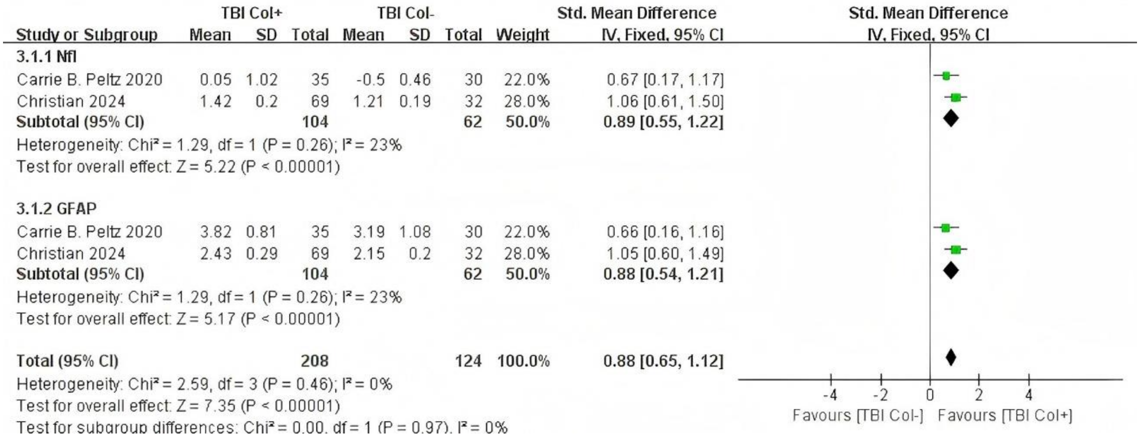

Supplementary Figure S1 The meta-analysis of blood NfL and GFAP concentrations in TBI CoI+ group and TBI CoI- group

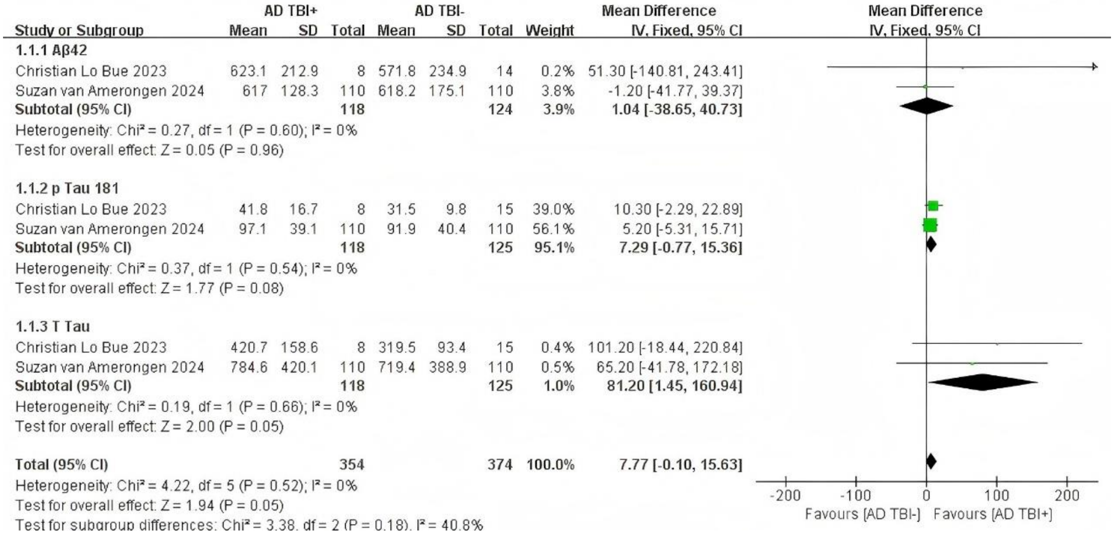

Supplementary Figure S2 The meta-analysis of CSF markers in AD TBI+ group and AD TBI- group

References

[1] Asken BM, Tanner JA, Gaynor LS, et al. Alzheimer's pathology is associated with altered cognition, brain volume, and plasma biomarker patterns in traumatic encephalopathy syndrome. Alzheimers Res Ther. 2023. 15(1): 126.

[2] Graham N, Zimmerman KA, Moro F, et al. Axonal marker neurofilament light predicts long-term outcomes and progressive neurodegeneration after traumatic brain injury. Sci Transl Med. 2021. 13(613): eabg9922.

[3] Lippa SM, Gill J, Brickell TA, Guedes VA, French LM, Lange RT. Blood Biomarkers Predict Future Cognitive Decline after Military-Related Traumatic Brain Injury. Curr Alzheimer Res. 2022. 19(5): 351-363.

[4] Bernick C, Shan G, Ritter A, et al. Blood biomarkers and neurodegeneration in individuals exposed to repetitive head impacts. Alzheimers Res Ther. 2023. 15(1): 173.

[5] Shahim P, Politis A, van der Merwe A, et al. Time course and diagnostic utility of NfL, tau, GFAP, and UCH-L1 in subacute and chronic TBI. Neurology. 2020. 95(6): e623-e636.

[6] Newcombe V, Ashton NJ, Posti JP, et al. Post-acute blood biomarkers and disease progression in traumatic brain injury. Brain. 2022. 145(6): 2064-2076.

[7] van Amerongen S, Das S, Kamps S, et al. Cerebrospinal fluid biomarkers and cognitive trajectories in patients with Alzheimer's disease and a history of traumatic brain injury. Neurobiol Aging. 2024. 141: 121-128.

[8] Shahim P, Pham DL, van der Merwe AJ, et al. Serum NfL and GFAP as biomarkers of progressive neurodegeneration in TBI. Alzheimers Dement. 2024. 20(7): 4663-4676.

[9] Jia X, Li X, Ji Q, et al. Serum biomarkers and disease progression in CT-negative mild traumatic brain injury. Cereb Cortex. 2024. 34(1): bhad405 [pii].

[10] Ni P, Qiao Y, Tong W, Zhao C, Zheng P. Associations between serum tau, neurological outcome, and cognition following traumatic brain injury. Neurol India. 2020. 68(2): 462-467.

[11] Clark AL, Weigand AJ, Bangen KJ, et al. Higher cerebrospinal fluid tau is associated with history of traumatic brain injury and reduced processing speed in Vietnam-era veterans: A Department of Defense Alzheimer's Disease Neuroimaging Initiative (DOD-ADNI) study. Alzheimers Dement (Amst). 2021. 13(1): e12239.

[12] Dey S, Gangadharan J, Deepika A, et al. Correlation of ubiquitin C terminal hydrolase and S100β with cognitive deficits in young adults with mild traumatic brain injury. Neurol India. 2017. 65(4): 761-766.

[13] Howard E, Moody JN, Prieto S, Hayes JP, Alzheimer’s Disease Neuroimaging Initiative, . Higher Cerebrospinal Fluid Levels of Amyloid-β40 Following Traumatic Brain Injury Relate to Confrontation Naming Performance. J Alzheimers Dis. 2024. 100(2): 539-550.

[14] Slavoaca D, Birle C, Stan A, et al. Prediction of Neurocognitive Outcome after Moderate-Severe Traumatic Brain Injury Using Serum Neuron-Specific Enolase and S100 biomarkers. J Med Life. 2020. 13(3): 306-313.

[15] Failla MD, Juengst SB, Arenth PM, Wagner AK. Preliminary Associations Between Brain-Derived Neurotrophic Factor, Memory Impairment, Functional Cognition, and Depressive Symptoms Following Severe TBI. Neurorehabil Neural Repair. 2016. 30(5): 419-30.

[16] Jin G, Yang Y, Bi F, Yang M, Ma Y. 5-HT and S100β values in evaluating severity of cognitive impairment after traumatic brain injury. Folia Neuropathol. 2023. 61(1): 47-52.

[17] Milleville KA, Awan N, Disanto D, Kumar RG, Wagner AK. Early chronic systemic inflammation and associations with cognitive performance after moderate to severe TBI. Brain Behav Immun Health. 2021. 11: 100185.

[18] Samatra D, Pratiwi N, Widyadharma I. High Il-1β Serum as a Predictor of Decreased Cognitive Function in Mild Traumatic Brain Injury Patients. Open Access Maced J Med Sci. 2018. 6(9): 1674-1677.

[19] Sun Y, Bai L, Niu X, et al. Elevated Serum Levels of Inflammation-Related Cytokines in Mild Traumatic Brain Injury Are Associated With Cognitive Performance. Front Neurol. 2019. 10: 1120.

[20] Eagle SR, Sherry N, Kershaw EE, et al. Impact of obese body mass index on inflammasome blood biomarkers and neurocognitive performance following traumatic brain injury with Glasgow coma scale 13 to 15. J Neurol Sci. 2024. 464: 123159.

[21] Su SH, Xu W, Li M, et al. Elevated C-reactive protein levels may be a predictor of persistent unfavourable symptoms in patients with mild traumatic brain injury: a preliminary study. Brain Behav Immun. 2014. 38: 111-7.
